# Microbiological profile and multidrug resistant bacteria in pneumonia

**DOI:** 10.1186/1471-2334-13-S1-O13

**Published:** 2013-12-16

**Authors:** Adriana Slavcovici, Cristina Cismaru, Mihaela Sabou, Mirela Flonta, Natalia Hagău

**Affiliations:** 1 Department of Infectious Diseases, Iuliu Hațieganu University of Medicine and Pharmacy, Clinical Hospital of Infectious Diseases, Cluj-Napoca, Romania; 2 Clinical Laboratory, Clinical Hospital of Infectious Diseases, Cluj-Napoca, Romania; 3 Department of Anesthesiology and Intensive Therapy, Iuliu Hațieganu University of Medicine and Pharmacy, University Emergency County Hospital of Cluj, Cluj-Napoca, Romania

## Background

Currently accepted classifications of pneumonia include community acquired pneumonia (CAP), healthcare-associated pneumonia (HCAP), hospital-acquired pneumonia (HAP), and ventilator-associated pneumonia (VAP). Previous studies have shown a high rate of patients with HCAP or HAP caused by multidrug-resistant bacteria (MDR). Our aim was to determine the differences in etiology and antibiotic-resistant bacteria between CAP, HCAP and HAP groups.

## Methods

We performed a retrospective study in which we included patients over 18 years old, with culture-positive pneumonia, between 2008 and 2011, from 3 clinical hospitals. The patients were classified as having CAP, HCAP or HAP. We recorded the bacteriologic results for the following samples: blood culture, sputum, bronchial aspirate, bronchoalveolar lavage. The statistical analysis was carried out using Graph Pad Prism 5.

## Results

A total of 340 patients were recorded (160 with CAP, 39 with HCAP and 141 with HAP). *Streptococcus pneumoniae* and *Haemophilus influenzae* were seen more frequently in CAP (33%, 14.4%) than in HCAP or HAP (1.4%, 0%; p<0.0001). The most common pathogens in HAP were MRSA (27.7%), *Acinetobacter* spp. (26.2%), *Klebsiella pneumoniae* (19%). Compared to the HCAP, *Acinetobacter* spp. and MRSA were significantly associated with HAP (OR 54.8, p<0.0001, LR 3.9). Compared to the HAP *P aeruginosa* and *E coli* were more common in HCAP (36%, p 0.04, OR 2; respectively 23%, p 0.001, OR 7). Among Gram-negative bacilli, resistance to ceftazidime was higher (86%) and significantly associated with HAP (OR 14.3, p<0.0001, LR 2.7). The extended-spectrum β-lactamase-producing *Enterobacteriaceae*, were more frequent in HAP (67.7%). However, no differences were found regarding ESBL producing *Enterobacteriaceae* between HAP and HCAP (p=0.06). MDR bacteria, including MRSA and gram-negative bacilli, were identified more frequently in HAP versus HCAP (78.7% vs. 12.8%; p<0.0001, OR 25, LR 6).

## Conclusion

*S pneumoniae* was the main causative pathogen in CAP. Despite the higher frequency of *P aeruginosa* in the HCAP group, the MDR bacteria were associated with the HAP group. Our results reveal important differences between HAP and HCAP concerning the etiology and antibiotic resistance patterns.

